# OXPHOS-Mediated Induction of NAD^+^ Promotes Complete Oxidation of Fatty Acids and Interdicts Non-Alcoholic Fatty Liver Disease

**DOI:** 10.1371/journal.pone.0125617

**Published:** 2015-05-01

**Authors:** Thomas E. Akie, Lijun Liu, Minwoo Nam, Shi Lei, Marcus P. Cooper

**Affiliations:** Division of Cardiovascular Medicine, Department of Medicine, University of Massachusetts Medical School, Worcester, Massachusetts 01605, United States of America; University of Basque Country, SPAIN

## Abstract

OXPHOS is believed to play an important role in non-alcoholic fatty liver disease (NAFLD), however, precise mechanisms whereby OXPHOS influences lipid homeostasis are incompletely understood. We previously reported that ectopic expression of LRPPRC, a protein that increases cristae density and OXPHOS, promoted fatty acid oxidation in cultured primary hepatocytes. To determine the biological significance of that observation and define underlying mechanisms, we have ectopically expressed LRPPRC in mouse liver in the setting of NAFLD. Interestingly, ectopic expression of LRPPRC in mouse liver completely interdicted NAFLD, including inflammation. Consistent with mitigation of NAFLD, two markers of hepatic insulin resistance—ROS and PKCε activity—were both modestly reduced. As reported by others, improvement of NAFLD was associated with improved whole-body insulin sensitivity. Regarding hepatic lipid homeostasis, the ratio of NAD^+^ to NADH was dramatically increased in mouse liver replete with LRPPRC. Pharmacological activators and inhibitors of the cellular respiration respectively increased and decreased the [NAD^+^]/[NADH] ratio, indicating respiration-mediated control of the [NAD^+^]/[NADH] ratio. Supporting a prominent role for NAD^+^, increasing the concentration of NAD^+^ stimulated complete oxidation of fatty acids. Importantly, NAD^+^ rescued impaired fatty acid oxidation in hepatocytes deficient for either OXPHOS or SIRT3. These data are consistent with a model whereby augmented hepatic OXPHOS increases NAD^+^, which in turn promotes complete oxidation of fatty acids and protects against NAFLD.

## Introduction

Non-alcoholic fatty liver disease (NAFLD) is rapidly becoming one of the leading causes of liver disease in Western societies [1]. NAFLD spans a spectrum of disease. In simple steatosis, there is benign accumulation of hepatic lipid [2]. In steatohepatitis, inflammation and attendant hepatocyte injury occur, which together may progress to fibrosis or, on rare occasion, hepatocellular carcinoma [3,4]. Worldwide, the prevalence of fatty liver disease is 10–30% [5,6]. Obesity and diabetes constitute key factors in its development. Recent work has demonstrated that, in NAFLD, hepatic insulin resistance exacerbates whole body insulin resistance [7]. Efforts to mitigate NAFLD are, thus, anticipated to improve not only fatty liver disease but also mitigate whole body insulin resistance and diabetes. Central to this goal is deciphering the key pathways that influence non-alcoholic fatty liver.

Defective mitochondrial oxidative phosphorylation (OXPHOS) features prominently in NAFLD. There is a striking negative association between OXPHOS and NAFLD. Notably, OXPHOS activity is decreased in patients with non-alcoholic fatty liver disease [8,9]. Functional changes are supported by energetically unfavorable perturbations in mitochondrial cristae, which are the structural underpinning of OXPHOS [10,11]. These changes are accompanied by both activation of protein kinase C epsilon (PKCε) via increased diacylglycerol (DAG) and an increase in mitochondrial reactive oxygen species (ROS) [12,13]. Both pathways have been implicated in hepatic insulin resistance, and thus, proposed as important mediators of hepatic insulin resistance in NAFLD. Additionally, fatty liver associates with reductions in OXPHOS-dependent [NAD^+^]/[NADH], which allosterically regulates complete oxidation of fatty acids via the citric acid cycle [14,15]. Due to a number of confounding variables, a causal role for defective OXPHOS in NAFLD and putative underlying mechanisms remains obscure. Hitherto, efforts to increase OXPHOS in liver have been thwarted by a lack of genetic (or pharmacological) tools that specifically augment hepatic OXPHOS *in vivo*. Hence, the precise role of augmented OXPHOS in NAFLD remains an open question.

Here we activate OXPHOS in liver and show that it interdicts NAFLD and mitigates whole body insulin resistance. We increased OXPHOS in mouse liver by ectopically expressing leucine-rich pentatricopeptide repeat containing (LRPPRC; but also called LRP130). LRPPRC is a 130 kD protein that is defective in Leigh Syndrome French Canadian variant, a rare form of the mitochondrial disorder Leigh Syndrome [16]. In both gain- and loss-of-function models, LRPRPC regulates mitochondrially encoded gene expression, which in turn regulates the expression of 13 polypeptides central to the formation of mature OXPHOS complexes [17–23]. LRPPRC is reported to regulate mitochondrially encoded gene expression by increasing mitochondrial transcription [18–20] as well as stabilizing some mitochondrially encoded transcripts [22,24]. Because an increase in mitochondrially encoded subunits is sufficient to drive mature OXPHOS complexes, ectopic expression of LRPPRC increases mitochondrial OXPHOS and oxygen consumption [18,19]. Using mice with transgenic expression of LRPPRC in liver, we show enhanced hepatic OXPHOS protects against fatty liver and reduces liver inflammation in the setting of diet-induced obesity. Additionally, hepatic and whole-body insulin sensitivity were increased, changes possibly explained by reduced reactive oxygen species and diminished PKCε activation. Mice with enhanced hepatic OXPHOS have a dramatic increase in the ratio of [NAD^+^]/[NADH], which promotes complete lipid oxidation in hepatocytes. Using pharmacological activators and inhibitors of cellular respiration, we demonstrate that respiratory capacity dictates [NAD^+^]/[NADH] in hepatocytes. Moreover, increasing the concentration of NAD^+^ in hepatocytes promotes complete fatty acid oxidation, and rescues impaired fatty acid oxidation in hepatocytes deficient for either OXPHOS or SIRTUIN 3 (SIRT3). Taken together, these data suggest that augmented hepatic OXPHOS enhances NAD^+^, which in turn promotes complete fatty acid oxidation and interdicts NAFLD.

## Materials and Methods

### Animal Model

Liver-specific *Lrpprc* transgenic mice were created as described previously [18]. Liver-specific expression was driven by a transthyretin enhancer and promoter. These mice were backcrossed to a C57BL/6 background for at least 6 generations. Wild-type littermate control mice were used to make comparisons. Mice were housed in a facility with 12h light/12h dark cycle. For high-fat diet feeding, male mice aged 7 weeks were fed a 55% kcal from fat diet (Harlan Teklad TD-93075) for 12 weeks before metabolic phenotyping. Following the designated high-fat feeding period, mice were sacrificed by CO_2_ euthanasia and tissues embedded for histology or flash-frozen in liquid nitrogen for further analysis. All animal experiments were approved by the IACUC of the University of Massachusetts Medical School (Protocol #A-2085-12).

### Pathological Assessment of Liver

Hepatic steatosis and inflammation in liver sections was assessed by an unbiased pathologist as previously described [25]. Briefly, steatosis was graded as Healthy (<5% of liver involvement), Mild (5–33%), Moderate (34–66%) or Severe (>66%), and inflammation as Healthy (no inflammation), Mild (1–2 foci per 10X field), Moderate (2–4 foci), or Severe (>4 foci).

### Metabolic Cage Analysis

Analysis of energy expenditure, activity, and food intake was performed by the University of Massachusetts Mouse Metabolic Phenotyping Core using metabolic cages (TSE Systems).

### Protein and Gene Expression

Whole tissue lysates were harvested by homogenization in a TissueLyzer (Qiagen) using lysis buffer (50mM Tris pH 7.5, 0.15mM NaCl, 1% NP-40, 0.25% sodium deoxycholate, 1mM EDTA) including a protease inhibitor cocktail (Sigma) and separated by SDS-PAGE (Invitrogen). Samples were transferred to a PVDF membrane and blocked in 5% BSA solution for 1 hour, then incubated with primary antibody overnight at 4°C. Membranes were washed three times with tris-buffered saline (50mM Tris, 150mM NaCl, 0.1% Tween-20, pH 7.6) then probed with the appropriate secondary antibody for 1 hour at room temperature. Following secondary incubation, samples were washed as above and proteins detected using ECL (GE Healthcare). Antibodies to LRPPRC were generated as previously described [18]. Antibodies for Myc, Actin (Santa Cruz), COX1, Citrate Synthase, VDAC (MitoSciences), phospho-AKT, AKT (Cell Signaling) were purchased and diluted according to the manufacturer instructions. Densitometric analysis was performed using ImageStudio (Licor) by comparison to a reference protein (actin). RNA samples were harvested from tissue in Trizol (Invitrogen) and analyzed as described previously [18].

### Mitochondrial Respiration, Complex Activity, and Isolation of Primary Hepatocytes

Mitochondria were harvested from chow-fed WT and Tg/Tg mouse livers and oxygen consumption in the presence of pyruvate and malate measured on a Clarke-type electrode (Hansatech) as previously described [26] using 0.5 mg of mitochondrial protein. All mitochondria used had an RCR > 3.0 and membrane integrity > 80%. Individual respiratory complex activity was performed as previously described [18]. Primary hepatocytes from WT and *Lrpprc* transgenic mice were isolated by perfusion and collagenase treatment as previously described [27].

### Measurement of Liver and Serum Triglycerides

Triglyceride levels in serum and liver from mice fasted for 16 hours were measured by PicoProbe fluorometric assay (BioVision) according to the manufacturer’s specifications. For liver, ~10 mg of tissue was homogenized in 5% Triton X-100 in water using a TissueLyzer (Qiagen) then boiled twice for 5 minutes to solubilize lipids. Samples were spun 13,000 x g for 5 minutes to pellet insoluble materials, then assayed using the provided materials. Values were normalized to protein concentration as measured by BCA assay (Thermo).

### Glucose and Insulin Tolerance Testing

Glucose tolerance testing was performed after an overnight fast by intraperitoneal injection of 1.5 mg/g dextrose. Blood glucose content was analyzed at 15, 30, 60, 90, and 120 minutes by tail vein sampling. For insulin tolerance, mice were fasted 6 hours, then injected with 0.75mU/g human insulin and monitored similarly. Blood glucose levels were determined using a OneTouch Ultra monitor and strips (LifeScan).

### Determination of Mitochondrial Superoxide, Proton Gradient, and PKCε Acvitiy

We used MitoSox Red (Invitrogen) per manufacturer protocol to measure mitochondrial superoxide in human hepatoma (HepG2) cells stably expressing ectopic human *Lrpprc*. Proton gradient was determined in cells plated in a 96-well dish by staining with 1μg/mL JC-1 dye (Life Technologies) for 20 minutes at 37°C per manufacturer instructions.

Cytosolic and membrane-bound PKCε was determined by fractionation and immunblotting as previously described [28]. The presence of PKCε was determined by immunoblot and activity calculated using the ratio of membrane to cytosolic signal as determined by densitometry.

### Nicotinamide Treatment and [NAD^+^]/[NADH] Measurement

Primary hepatocytes were incubated for 16 hours in serum-free medium in the presence or absence of nicotinamide (NAM) at the doses indicated. For inhibitor experiments, cells were incubated for 2 hours in the presence of 4 μM Carbonyl cyanide-4-(trifluoromethoxy)phenylhydrazone (FCCP) or 1 μM rotenone. Measurement of NAD^+^ and NADH was performed using the EnzyFluo [NAD^+^]/[NADH] kit (BioAssays) per the manufacturer’s instructions.

### Fatty Acid Oxidation Assay

The oxidation of palmitate was measured in primary hepatocytes as previously described with minor modifications [18]. Briefly, primary hepatocytes were incubated in serum-free medium containing 20nM glucagon ± NAM for 16 hours. Following this incubation, medium was changed to DMEM with 1% BSA Fraction V, 20 mM HEPES, and 250 μM cold palmitate. Following incubation at 37°C for 2 hours, wells were spiked ^14^C-palmitic acid and incubated for an additional 90 minutes. Complete oxidation of palmitate was measured by the release of ^14^C-carbon dioxide after addition of perchloric acid (10% v/v final concentration). Chemical inhibition of OXPHOS was accomplished by treatment with either 4 μM FCCP or 1 μM rotenone 30 minutes prior to the addition of radiolabeled substrate.

### Statistics

Statistical analyses were performed using GraphPad (Prism) or SAS/STAT. Differences in protein densitometry, cellular respiration, relative mtDNA levels, area-under-the-curve for glucose and insulin tolerance tests, triglyceride levels, and mitochondrial membrane potential were compared by two-tailed, unpaired Student’s t-test. Membrane-bound and membrane-to-cytosol ratios of PKCε were compared using a one-tailed, unpaired Student’s t-test. Gene expression was analyzed by either two-tailed, unpaired Student’s t-test (nuclear encoded transcription factors) or by two-way ANOVA using Bonferroni’s post test. Comparison of maximal respiration to LRPPRC protein levels was performed using linear regression. Steatosis and inflammation grading was assessed by chi-square analysis. For determination of differences in mouse weight curves, a mixed model with Bonferroni correction was performed. In general, *P* values less than 0.05 were considered significant.

## Results

### Ectopic expression of liver-specific LRPPRC promotes hepatic OXPHOS

**Fig 1:**
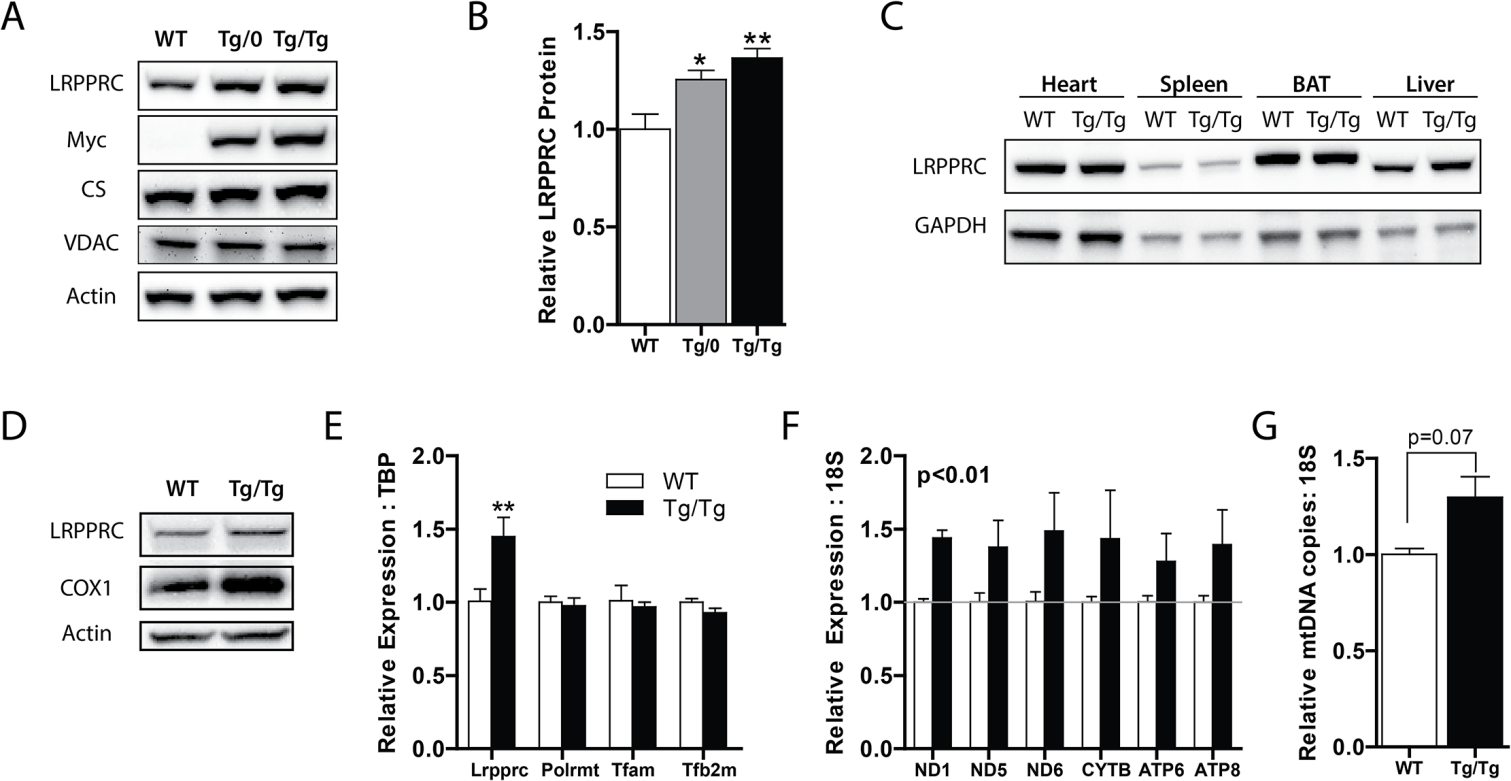
LRPPRC promotes mitochondrially encoded gene expression in liver. (A) Protein expression and (B) quantification in livers of wild-type littermate controls (WT), hemizygous (Tg/0), and double hemizygous (Tg/Tg) LRPPRC transgenic mice. (C) Protein expression in heart, spleen, brown adipose tissue (BAT) and liver in WT and Tg/Tg mice. (D) Protein expression in WT and Tg/Tg cultured primary hepatocytes. (E) Nuclear *Lrpprc*, mitochondrial polymerase (*Polrmt*), mitochondrial transcription factor A (*Tfam*), and mitochondrial transcription factor B2 (*Tfb2m*) and (F) mitochondrially encoded respiratory complex subunit gene expression in Tg/Tg and control livers. (G) Genetic determination of mitochondrially encoded DNA content in the samples. For all experiments, n = 3 or 4. Data are mean ± SEM * p<0.05, ** p<0.01, *** p<0.001 vs. WT by two-tailed unpaired Student’s t-test (B, E, G) or 2-way ANOVA with Bonferroni’s post-test (F).

In order to assess the role of OXPHOS in the progression of NAFLD *in vivo*, we ectopically expressed *Lrpprc* (also called *Lrp130*) in a liver-specific manner. As shown in Fig 1A and 1B, there is a dose-dependent increase of LRPPRC protein in liver harvested from hemizygous (Tg/0) and double-hemizygous transgenic mice (Tg/Tg). Gene expression for *Lrpprc* matched LRPPRC protein levels (Fig 1E and S1 Fig). Importantly, ectopic expression of LRPPRC protein was not observed in other tissues, supporting liver-specific expression (Fig 1C). Because double transgenic mice had the greatest expression of LRPPRC protein, and we sought to maximize our model of hepatic OXPHOS, double hemizygous mice became the major focus of our studies, while hemizygous mice were used in select experiments. Throughout our studies, the phenotype of hemizygous mice was intermediate between wild-type littermate control mice and double hemizygous transgenic mice. Consistent with prior reports, ectopically expressed LRPPRC resulted in increased mitochondrially encoded gene expression (Fig 1F and S1 Fig). Additionally, protein analysis in primary hepatocytes isolated from Tg/Tg mice showed greater COX1 protein levels compared with WT mice (Fig 1D). We did not observe changes in several markers of mitochondrial content—mRNA expression of *Polrmt*, *Tfam*, and *Tfb2m* (Fig 1E), protein expression of citrate synthase or VDAC (Fig 1A), or mtDNA copy number (Fig 1G), suggesting augmentation of OXPHOS was not due to increased mitochondrial mass.

**Fig 2:**
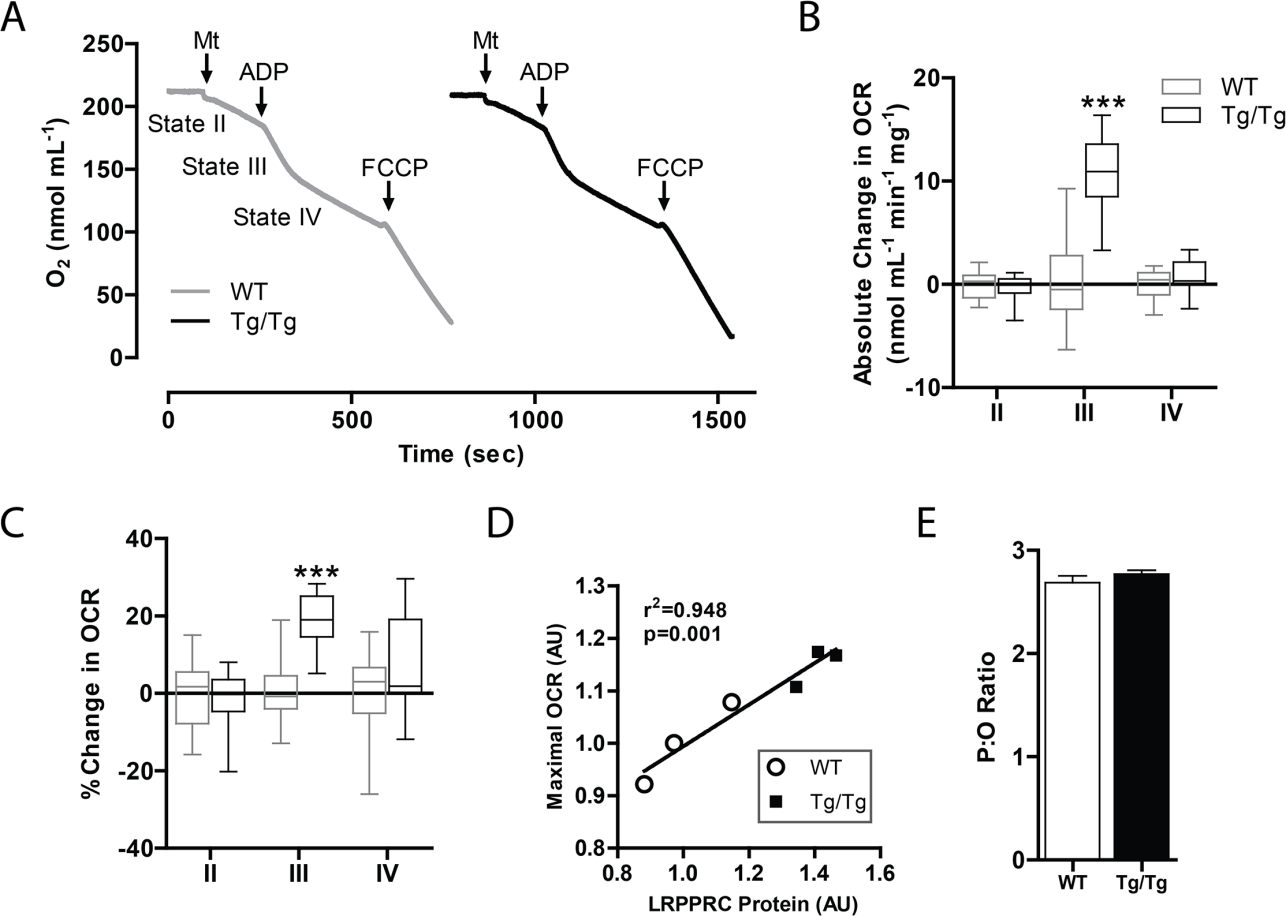
Liver-specific LRPPRC augments hepatic OXPHOS. (A) Representative plot, (B) absolute and (C) relative changes in oxygen consumption rate (OCR) in isolated mitochondria from WT and LRPPRC Tg/Tg livers measured on a Clarke-type electrode. (D) FCCP induced maximal OCR vs. LRPPRC protein levels in isolated mitochondria from WT and Tg/Tg livers. (E) P:O ratio calculated from Clarke-electrode data. For all experiments, n = 3 or 4. Data are mean ± SEM (B—E), * p<0.05, ** p<0.01, *** p<0.001 by two-tailed unpaired Student’s t-test (B, C) or linear regression (D).

Next, we tested if ectopic expression of hepatic LRPPRC could augment OXPHOS in mouse liver mitochondria. Using mitochondria isolated from livers of chow fed wild-type littermate control mice and Tg/Tg mice, we measured oxygen consumption using a Clarke-type electrode (Fig 2A). While basal (state II and IV) respiration was unchanged in transgenic mitochondria, ADP-stimulated respiration (state III) was increased (Fig 2B and 2C). Furthermore, the level of LRPPRC protein predicted maximal oxygen consumption (FCCP) in both WT and Tg/Tg mitochondria (r^2^ = 0.948, p = 0.001), indicating that respiratory capacity directly correlates with LRPPRC protein expression (Fig 2D). Compared with WT mitochondria, Tg/Tg mitochondria had equivalent levels of proton leak (data not shown), indicating that the increase in oxygen consumption was attributable to *bona fide* OXPHOS. These functional measurements are in agreement with observed increases in complex I, III, IV and V activities for cells replete with LRPPRC (S2 Fig and ref. [18]).

Increased oxygen consumption could result from enhanced efficiency of OXPHOS or an increase in the number of OXPHOS units per mitochondrion, that is, cristae density. As shown in Fig 2E, the P:O ratio (that is, the ratio of ATP molecules generated per molecule of oxygen consumed in state III) was unaltered in transgenic mitochondria, indicating increased OXPHOS units per mitochondrion. While we did not measure cristae density in this study, these data are consistent with previous reports by our group and others demonstrating a role for LRPPRC in regulating mitochondrial cristae density [18,21]. In summary, ectopic expression of LRPPRC in the liver augments hepatic OXPHOS without gross alterations in mitochondrial mass.

### Augmented hepatic OXPHOS improves whole body insulin sensitivity

**Fig 3:**
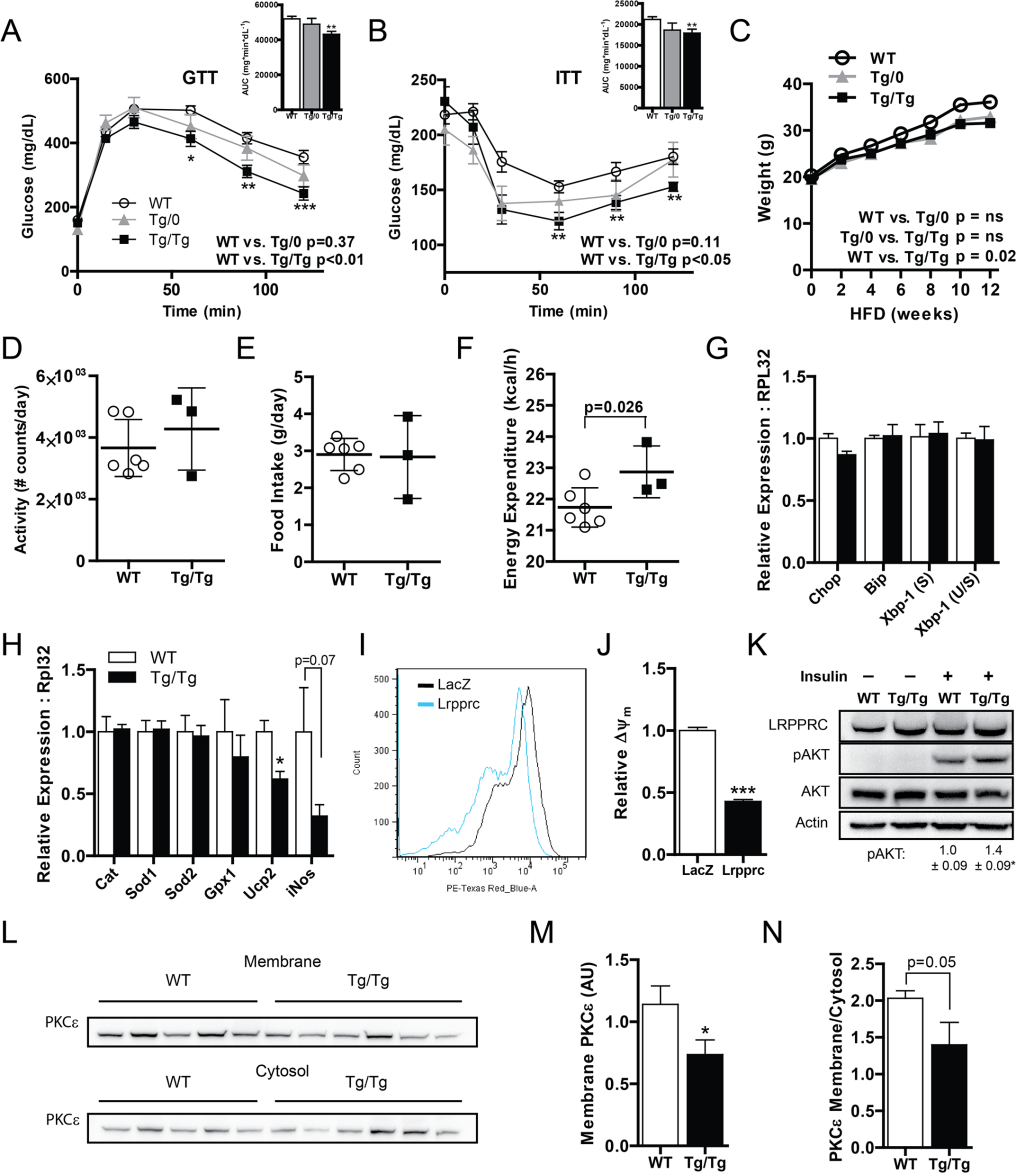
Hepatic OXPHOS enhances whole body insulin sensitivity. (A) Glucose and (B) insulin tolerance tests and area-under-the-curve (insets) and (C) weights in wild-type littermate control mice (WT), LRPPRC single hemizygous (Tg/0), and double hemizygous (Tg/Tg) mice fed a high-fat diet for 12 weeks, n = 10. (D) Hourly activity, (E) daily food intake, and (F) diurnal energy expenditure (7AM—7PM) in WT and Tg/Tg mice fed a high-fat diet > 20 weeks n = 3–6. (G) ER stress and (H) antioxidant gene expression in high-fat fed WT and Tg/Tg mouse liver, n = 9 or 10. (I) Mitochondrial superoxide production and (J) mitochondrial membrane potential (ΔΨ_m_) in HepG2 cells ectopically expressing LacZ (control) or LRPPRC n = 24. (K) Immunoblot and quantification of phospho-AKT in cultured primary hepatocytes from chow-fed WT and Tg/Tg mice treated with 25nM insulin, n = 3. (L) Immunoblot and (M) quantification of membrane or (N) membrane: cytosolic PKCε in high-fat fed WT and Tg/Tg mouse livers, n = 5 or 6. Data are mean ± SEM, *p<0.05, **p<0.01, ***p<0.001 vs. WT by two-way ANOVA (A, B), two-tailed unpaired Student’s t-test (insets, F, H, J, K), mixed model with Bonferroni correction (C) or one-tailed unpaired Student’s t-test (M, N).

The liver plays an important role in regulating whole body metabolic homeostasis. Therefore, we characterized the effect of enhanced hepatic OXPHOS on whole-body energy balance and insulin sensitivity. On a chow diet, WT,Tg/0, and Tg/Tg mice had no significant differences in weight or whole body insulin sensitivity as assessed by ITT or GTT (data not shown). In order to determine the role of increased hepatic OXPHOS in NAFLD, we induced fatty liver by feeding mice a high-fat diet (HFD; 55% kcal fat) for 12 weeks. Expression of *Lrpprc* and mitochondrially encoded OXPHOS genes remained elevated in Tg/Tg mice following high-fat feeding (S3 Fig). As shown in Fig 3A and 3B, mice with augmented hepatic OXPHOS had improved insulin sensitivity as assessed by ITT and GTT. Improvement in insulin sensitivity was dependent on the dose of LRPPRC, since double transgenic were more insulin sensitive than single transgenic liver-specific LRPPRC mice. Alterations in insulin sensitivity can be due to a multitude of factors, thus we evaluated processes that might explain this finding. Though weight gain was similar between all groups throughout most of the feeding timeline, there was a modest reduction in weight in Tg/Tg mice following 12-weeks of HFD (Fig 3C). Even so, there was a strong trend toward insulin sensitivity prior to changes in weight (data not shown), implying intrinsic effects independent of weight loss. To further evaluate this discrepancy in weight, we performed metabolic cage analysis of WT and Tg/Tg mice fed a HFD. Though there was no difference in daily activity or food intake (Fig 3D and 3E), mice with augmented hepatic OXPHOS tended to have increased diurnal energy expenditure (Fig 3F). We, therefore, screened several factors involved in whole body energy balance—hepatic *Fgf21*, hepatic *Il6*, and skeletal muscle *Irisin*; however, none of the mRNAs for these factors were altered (data not shown).

Since transgenic *Lrpprc* expression in our model is restricted to the liver (Fig 1C), we evaluated potential mechanisms by which augmented OXPHOS confers insulin sensitivity. Specifically we focused on ER stress, reactive oxygen species (ROS), and protein kinase C epsilon (PKCε) activation. As shown in Fig 3G, we saw no change in several mRNA known to correlate with ER stress. Regarding ROS, *Ucp2* and *iNOS*, which are markers of oxidative stress, were reduced in high-fat fed Tg/Tg livers (Fig 3H). Consistent with these results, ectopic *Lrpprc* expression in human hepatoma cells (HepG2 cell line) reduced mitochondrial superoxide production as assessed by MitoSox Red (Fig 3I). This indicates LRPPRC is sufficient to reduce ROS formation in a cell autonomous fashion. Mitochondrial production of ROS is, in part, dependent on inner membrane proton gradient [29]. Cells ectopically expressing LRPPRC had lower proton gradients compared with controls (Fig 3J), suggesting a likely mechanism for reduced mitochondrial superoxide. Because coupled respiration is increased (Fig 2), a reduced proton gradient could suggest enhanced proton pumping coupled with a disproportionately greater activity at complex V, which dissipates the proton gradient. This is consistent with previous work indicating differential effects of LRPPRC on Complex V [18,30].

To exclude weight reduction as a potential confounding factor in insulin resistance, we next evaluated insulin induced phosphorylation of AKT (pAKT) in primary hepatocytes isolated from mice on a chow fed diet. As shown in Fig 3K, primary hepatocytes from mice with augmented hepatic OXPHOS had modestly increased pAkt in response to insulin treatment. These data suggest two key findings. One, augmented hepatic OXPHOS modestly improves hepatocyte insulin signaling in a cell autonomous manner and lowers the mitochondrial proton gradient culminating in lower ROS. Two, improved hepatocyte signaling promotes whole body insulin signaling only in the setting of metabolic stress.

In fatty liver disease, increased triglyceride content promotes the accumulation of diacylglycerol (DAG) species, which in turn activates PKCε. Upon activation, PKCε translocates from the cytosol to the membrane, where it phosphorylates insulin receptor beta, culminating in impaired insulin signaling [31,32]. Assessment of membrane bound PKCε is, thus, a readout of PKCε activation and correlates with insulin resistance [33]. Activation of PKCε was reduced in mice with augmented hepatic OXPHOS (Fig 3L—3N). These data could imply decreased total lipid burden in liver with augmented OXPHOS.

### Augmented hepatic OXPHOS protects against NAFLD

**Fig 4:**
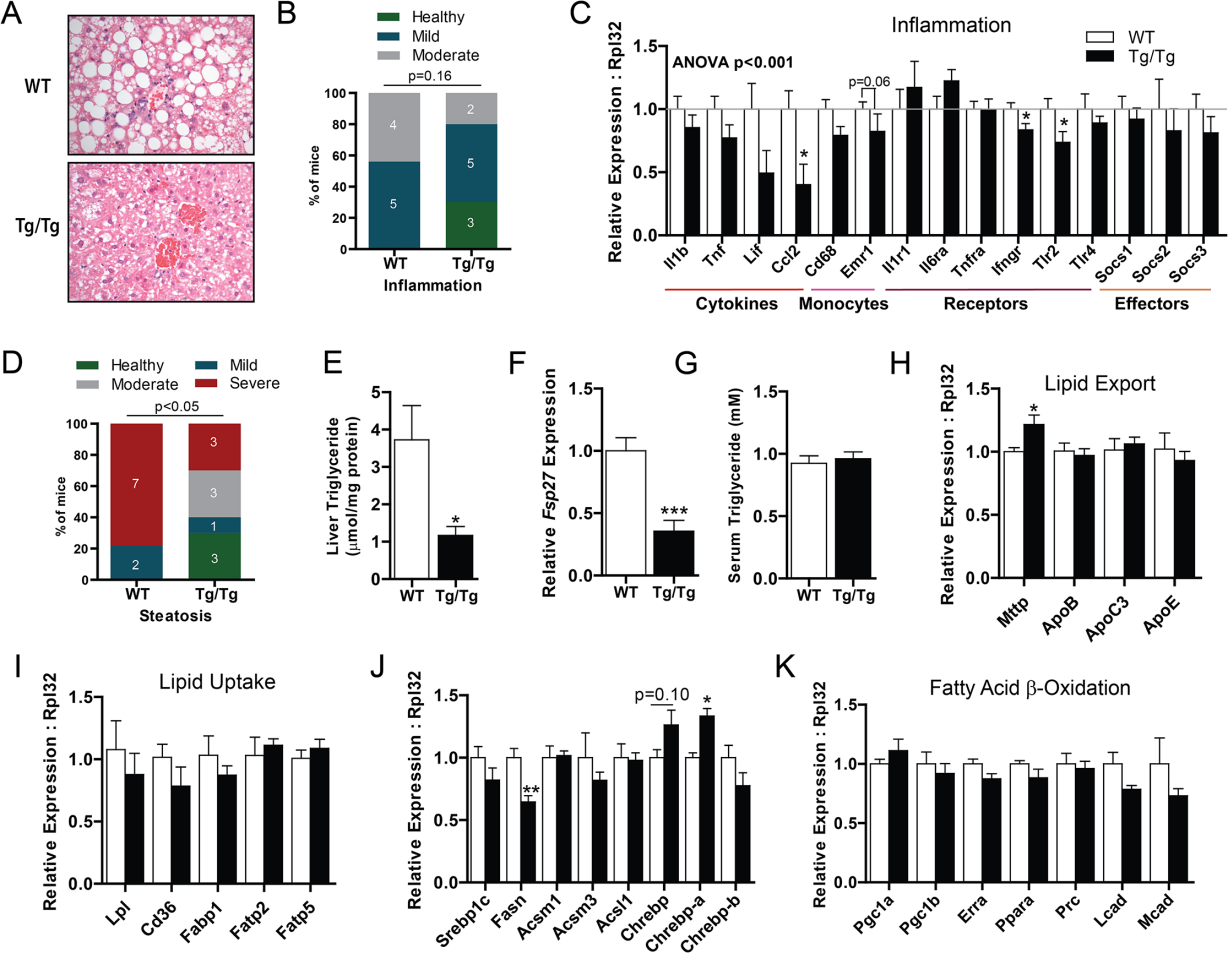
Augmented hepatic OXPHOS interdicts NAFLD. (A) Representative histological sections of livers from wild-type littermate control mice (WT) and LRPPRC double hemizygous (Tg/Tg) mice. (B) Pathologic classification of inflammation in livers of the same mice. (C) Expression of inflammatory genes in livers of WT and Tg/Tg mice fed a high-fat diet for 12 weeks. (D) Pathologic classification of steatosis in high-fat fed WT and Tg/Tg mouse livers. (E) Biochemical determination of triglyceride content in the same samples. (F) Determination of *Fsp27* expression in high-fat fed WT and Tg/Tg livers. (G) Serum triglyceride content in the same mice. Expression of genes involved in (H) lipid export, (I) lipid uptake, (J) Lipogenesis, and (K) fatty acid β-oxidation in high-fat fed WT and Tg/Tg mouse livers. For all experiments, n = 9 or 10. Data are mean ± SEM *p<0.05, **p<0.01, ***p<0.001 vs. WT by chi-squared analysis (B, D), 2-way ANOVA with Bonferroni’s post-test (C), or by two-tailed, unpaired Student’s t-test (D-K).

We hypothesized that enhanced OXPHOS might influence the course of NAFLD. We tested this hypothesis by feeding mice a HFD for 12 weeks to induce fatty liver disease. In mice, diet-induced NAFLD only results in modest inflammation. Nonetheless, this modest inflammation was reduced in transgenic mice compared with littermate control mice (Fig 4A and 4B, p = 0.16, chi-squared). In Tg/Tg mice, the frequency of mice with healthy livers was increased (30% Tg/Tg vs. 0% control). Similarly, the frequency of mice with moderate hepatic inflammation was reduced 2.2-fold (20% Tg/Tg mice vs. 44% control). In support of histological grading, the expression of panel of inflammatory genes was reduced in mouse liver from Tg/Tg mice (Fig 4C). Notably, *Ccl2* (also called *Mcp1*), a monocyte chemoattractant, was modestly reduced.

Next, we evaluated hepatic steatosis, using histological and biochemical assessments. Compared with WT littermate controls, Tg/0 and Tg/Tg mice show a dramatic reduction in hepatic lipid content (Fig 4A and S4 Fig). We also graded hepatosteatosis, using a well characterized grading system [25]. The frequency of mice with healthy livers was markedly increased in mice with augmented hepatic OXPHOS (30% Tg/Tg vs 0% control), and there was 2.6-fold reduction in steatotic livers (30% Tg/Tg vs. 78% control, Fig 4D). Consistent with these results, biochemical measurement of hepatic triglycerides and the expression of *Fsp27*, a gene that marks lipid size, were both reduced in livers from Tg/Tg mice (Fig 4E and 4F). This reduced lipid burden may explain diminished PKCε activation. Taken together, these data argue that augmentation of hepatic OXPHOS by ectopic expression of LRPPRC mitigates both steatosis and inflammation in NAFLD.

Regulation of hepatic lipid content is complex process that is dependent on integrated action of lipid transport, lipogenesis, and fatty acid oxidation. For example, an increase in hepatic lipid export can reduce hepatic steatosis but adversely increase serum triglycerides [34,35]. Similarly, a decrease in hepatic lipid uptake can reduce steatosis but adversely increase serum triglyceride. In mice with augmented hepatic OXPHOS, fasting serum triglycerides were not altered (Fig 4G), a finding which could imply that hepatic lipid export and import were not significantly altered. Consistent with this, genes involved in lipid transport were unchanged in Tg/Tg mice (Fig 4H and 4I).

Because hepatic lipogenesis is increased in patients with NAFLD [36], we quantified the expression of lipogenic genes. While *Srebp1c* was unchanged, *Fasn* mRNA was modestly reduced about 35% in Tg/Tg mice (Fig 4J). Even so, the difference in *Fasn* is unlikely to explain reduced hepatic steatosis in LRPPRC transgenic mice, since ablation of *Fasn* in mice paradoxically increases hepatic lipid content [37]. Additionally, we assessed the expression of *ChREBP* isoforms, a transcription factor which regulates *Fasn* and *Acc*. However, in Tg/Tg mice, total *ChREBP*, *ChREBP-a*, and *ChREBP-β* exhibited only minor changes compared to wild-type littermate controls.

We previously showed that ectopic Lrpprc expression promotes fatty acid β-oxidation in primary hepatocytes [18]. In this model, LRPPRC enhanced fatty acid oxidation without affecting the expression of genes involved in fatty acid β-oxidation. Consistent with our previous study, the expression of genes involved in fatty acid β-oxidation gene were unchanged in livers Tg/Tg mice (Fig 4K). These data imply metabolic control of fatty acid β-oxidation is critical.

### Augmented OXPHOS increases hepatic NAD^+^, which governs complete fatty acid oxidation

Status of hepatic OXPHOS directly correlates with fatty acid oxidation in liver. For example, in patients suffering from Leigh syndrome, which impairs OXPHOS, there is increased hepatic steatosis and reduced β-oxidative capacity [38,39]. Furthermore, pharmacological inhibition of OXPHOS impairs mitochondrial oxidation of fatty acids [40]. We previously published that ectopically expressing *Lrpprc* in hepatocytes increased OXPHOS as well as palmitate oxidation without alterations in the fatty acid oxidative program [18]. The absence of substantial genetic changes in these models of OXPHOS as well as in Tg/Tg mice (Fig 4K) could suggest a metabolic control of fatty acid oxidation. We, therefore, sought to investigate if metabolic control via augmented OXPHOS could promote complete fatty acid oxidation.

**Fig 5:**
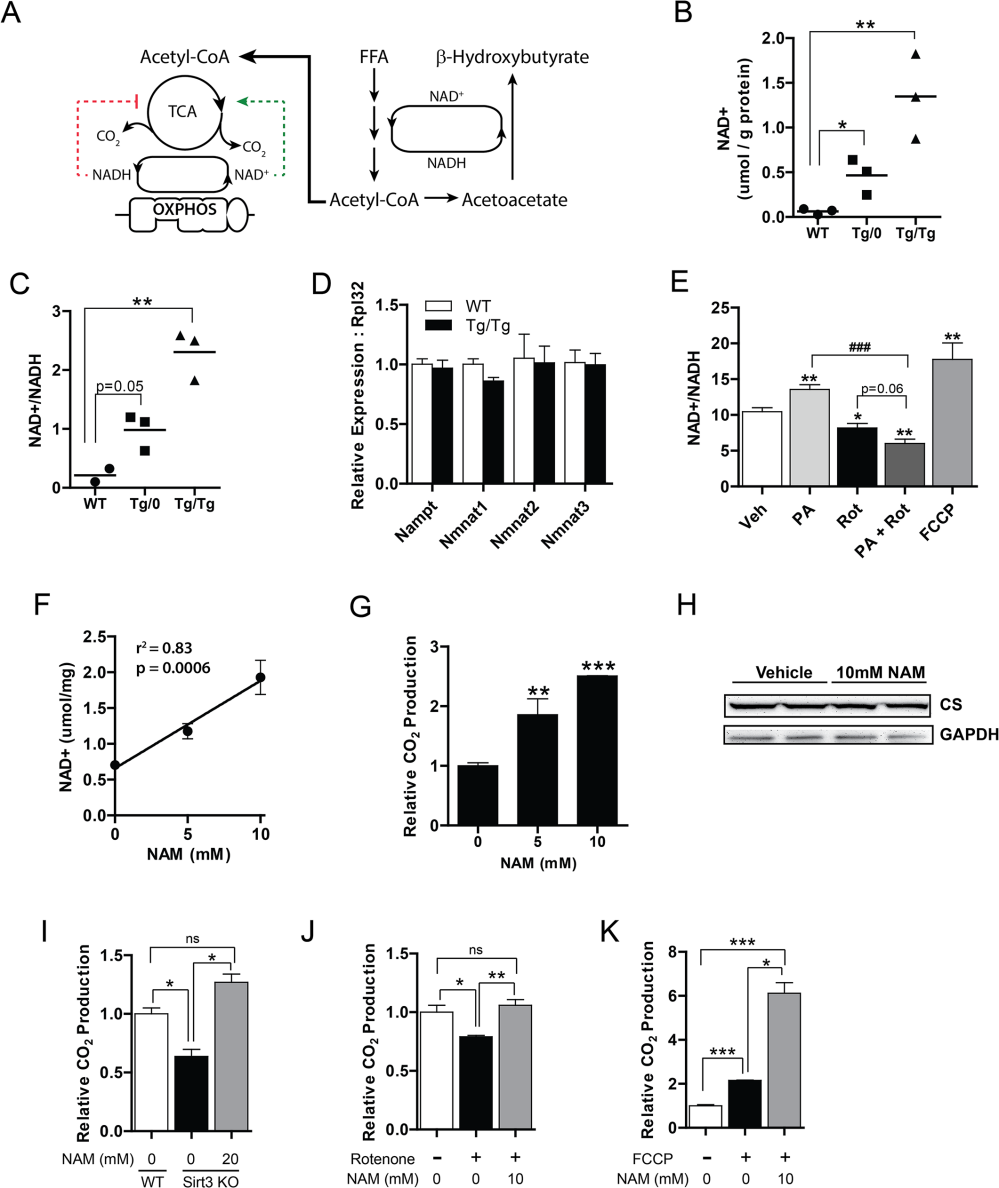
OXPHOS dictates NAD^+^, promoting complete oxidation of fatty acids (A) Fatty acid metabolic pathway in liver: NAD^+^ dependent β-oxidation catalyzes lipids to acetyl-CoA and ketone bodies (incomplete oxidation). Acetyl-CoA is further metabolized via the NAD^**+**^ dependent TCA cycle, yielding CO_2_ (complete oxidation). NAD^**+**^ allosterically activates TCA cycling, while NADH inhibits this process. (B) NAD^**+**^ and (C) [NAD^**+**^]/[NADH] levels in chow fed wild-type littermate control mice (WT), LRPPRC single hemizygous (Tg/0), and double hemizygous (Tg/Tg) mice.(D) NAD^**+**^ synthetic gene expression in WT and Tg/Tg mice. (E) [NAD^**+**^]/[NADH] levels in cultured primary hepatocytes treated with 250 μM palmitic acid, 1 μM rotenone, or 4 μM FCCP. (F) NAD^**+**^ levels in nicotinamide (NAM) treated cultured primary hepatocytes. (G) Palmitate oxidation to CO_2_ in cultured primary hepatocytes treated with NAM. (H) Immunoblot of citrate synthase (CS) expression in cultured primary hepatocytes treated with NAM. (I) Palmitate oxidation in *Sirt3* -/- (Sirt3 KO) primary hepatocytes treated with NAM. Palmitate oxidation in primary hepatocytes treated with (J) rotenone or (K) FCCP and NAM. For all experiments, n = 3 or 4 Data are mean ± SEM *p<0.05, **p<0.01, ***p<0.001 vs. vehicle unless otherwise indicated and ###p<0.001 by two-tailed unpaired Student’s t-test (B, C, E, G, I, J, K) or linear regression (F).

Complete oxidation of lipids requires oxidation of acetyl-CoA by the citric acid cycle, a process dependent on the oxidized electron carrier NAD^+^ (Fig 5A). Conversely, NADH opposes citric acid (TCA) cycle oxidation. The electron transport chain plays a central role in coenzyme concentration as it converts NADH to NAD^+^. Thus, we hypothesized that augmented OXPHOS might increase NAD^+^, which in turn might promote complete oxidation via the citric acid cycle. We measured NAD^+^ levels in mice with augmented OXPHOS fed a chow diet. As shown in Fig 5B and 5C, both Tg/0 and Tg/Tg mice had increased levels of NAD^+^ and the ratio of oxidized to reduced carriers ([NAD^+^]/[NADH]). We did not, however, observe any alteration in the rate limiting enzymes involved in NAD^+^ synthesis (Fig 5D).

Next, we confirmed these findings in a cellular model of hepatic OXPHOS. To exclude pleiotropic effects of LRPPRC expression, we measured the effects of chemical activators and inhibitors of cellular respiration on the ratio of [NAD^+^]/[NADH] in cultured primary mouse hepatocytes. To first test the effects of metabolic excess on [NAD^+^]/[NADH], we treated hepatocytes with palmitic acid. Consistent with previous work in hepatoma cell lines [41], palmitate enhanced [NAD^+^]/[NADH] in primary hepatocytes containing normal respiratory capacity (Fig 5E). We then wished to understand if modulation of cellular respiration altered [NAD^+^]/[NADH]. Inhibition of electron transport using the Complex I inhibitor rotenone significantly diminished [NAD^+^]/[NADH]. In contrast, increasing cellular respiration by the uncoupling agent FCCP enhanced [NAD^+^]/[NADH] levels. Since OXPHOS is disrupted in patients with NAFLD, we interrogated the consequences of nutrient excess in the context of a dysfunctional respiratory chain by measuring the availability of NAD^+^ in hepatocytes concurrently treated with palmitic acid and rotenone. As shown in Fig 5E, the addition of fatty acids synergistically diminished [NAD^+^]/[NADH] in hepatocytes treated with rotenone. This suggests that [NAD^+^]/[NADH] levels may only be disrupted by nutrient flux in the presence of impaired OXPHOS.

Given the availability of NAD^+^ is dictated by OXPHOS, and augmentated OXPHOS drives fatty acid oxidation, we evaluated whether increased NAD^+^ could was the basis for complete oxidation of fatty acids. To test this, we treated primary mouse hepatocytes with the NAD^+^ precursor nicotinamide (NAM). Treatment with NAM directly correlated with cellular NAD^+^ levels in cultured primary hepatocytes (Fig 5F). We next investigated if increased NAD^+^ promoted complete oxidation of fatty acids by measuring the complete oxidation of palmitate to CO_2_ in the presence of increasing NAM, which increases NAD^+^ levels. As shown in Fig 5G, NAM promoted a dose-dependent increase in complete palmitate oxidation to CO_2_, suggesting that NAD^+^ is capable of driving fatty acid oxidation. Importantly, we did not observe any effects of NAM on mitochondrial content as measured by the mitochondrial protein citrate synthase (Fig 5H).

Previous work indicates that NAD ^+^ activates the mitochondrial protein SIRT3, which deacetylates certain proteins involved in fatty acid β-oxidation and augments fatty acid oxidation [42]. We, therefore, assessed if the effects of NAM on palmitate oxidation were entirely dependent on SIRT3. Consistent with previously published data, primary hepatocytes lacking *Sirt3* (Sirt3 KO) had reduced fatty acid oxidative capacity when compared with wild-type hepatocytes (Fig 5I). Using NAM to increase NAD^+^, however, the deficit in Sirt3 KO hepatocytes was rescued. These data imply the effects of NAD^+^ on increasing complete fatty acid oxidation are independent of SIRT3 mediated deacetylation.

**Fig 6:**
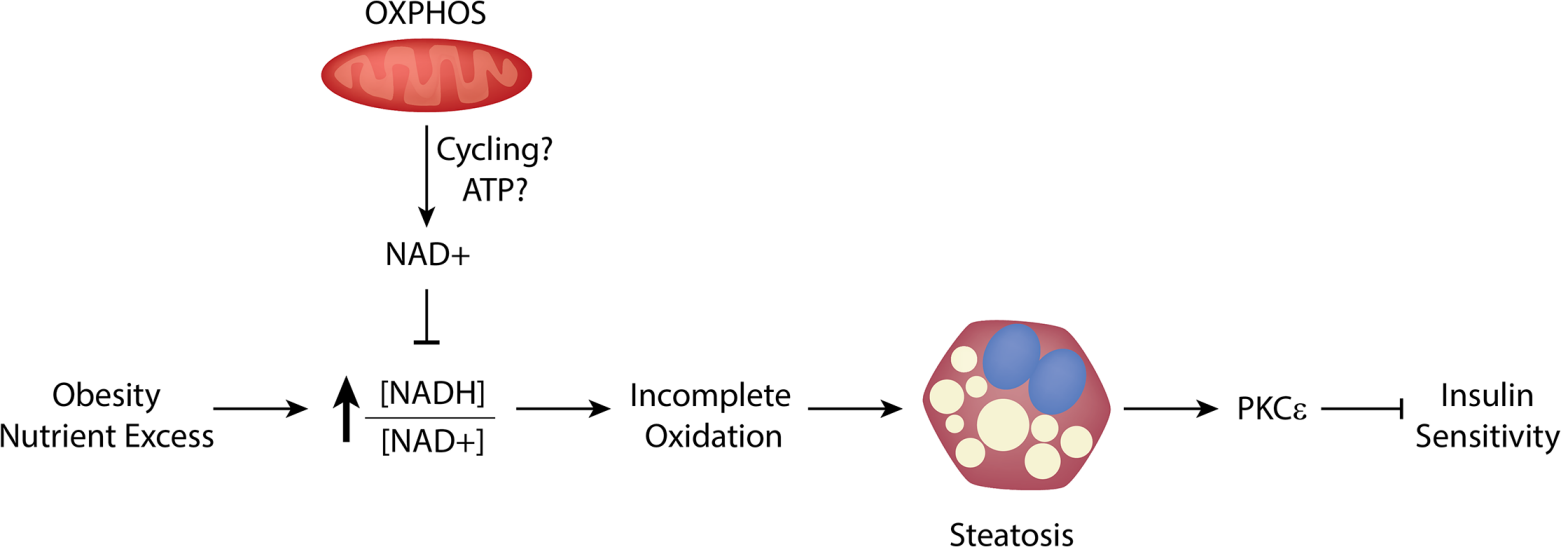
Activation of hepatic OXPHOS increases cellular NAD^+^ and complete fatty acid oxidation, mitigating NAFLD. In obesity and nutrient excess, increased ratio of reduced NADH to oxidized NAD^**+**^ inhibits complete oxidation of fatty acids in the liver, leading to lipid accumulation, PKCε activation, and insulin resistance. Augmented hepatic OXPHOS, through enhanced redox cycling and/or increased ATP dependent NAD^**+**^ synthesis, increases the ratio of [NAD^**+**^]/[NADH], thereby driving complete oxidation of fatty acids, reducing steatosis, and promoting insulin sensitivity. Additionally, enhanced OXPHOS reduces reactive oxygen species, which may contribute to increased insulin sensitivity (not shown).

We next sought to query the effects of exogenous NAD^+^ in hepatocytes with inhibited OXPHOS. To do so, we treated primary hepatocytes with rotenone, and measured complete oxidation of palmitate to carbon dioxide. Rotenone reduced palmitate oxidation by 20% compared with vehicle treated hepatocytes (Fig 5J). In the presence of NAM, however, complete fatty acid oxidation was rescued in OXPHOS deficient hepatocytes. Finally, we evaluated if NAD^+^ was capable of enhancing palmitate oxidation in the setting of augmented cellular respiration. We treated primary hepatocytes with the chemical uncoupler FCCP, which induces maximal respiration via uncoupling electron transport from ATP synthesis. FCCP driven maximal respiration promoted palmitate oxidation and increased CO_2_ production two-fold (Fig 5K). This increase was potentiated in cells treated with NAM, indicating that NAD^+^ augmented complete oxidation, even in the setting of increased cellular respiration. Taken together, these data are consistent with a model of OXPHOS driven NAD^+^, which promotes complete oxidation of lipids, which may explain reduced lipid accumulation in hepatocytes (Fig 6).

## Discussion

In this report, we demonstrated that increased hepatic oxidative phosphorylation (OXPHOS) increases NAD^+^, an allosteric activator of TCA cycle enzymes, which promotes complete oxidation of fatty acids. Consistent with a favorable change in hepatic lipid homeostasis through NAD^+^, mice with augmented hepatic OXPHOS had increased NAD^+^ and were protected from NAFLD. Increasing NAD^+^ in mouse hepatocytes rescued incomplete fatty acid oxidation in mice deficient for either OXPHOS or SIRT3 and synergistically increased complete oxidation of fatty acids in hepatocytes with increased cellular respiration. As reported by others, mitigation of NAFLD was associated with improved whole-body insulin sensitivity, presumably due to reduced liver inflammation and release of detrimental inflammatory cytokines. Overall, these data identify a critical role for OXPHOS-mediated control of NAD^+^ in NAFLD (Fig 6).

Several studies have demonstrated that the ratio of [NADH]/[NAD^+^] is increased in NAFLD [14,15]. Interestingly, in our studies, simply fluxing hepatocytes with palmitate did not did not increase the [NADH]/[NAD^+^] ratio. In the presence of impaired OXPHOS, however, the ratio was synergistically increased. This is important, since the ratio of [NADH]/[NAD^+^] directly correlates with mitochondrial reactive oxygen species (ROS), an important determinant of hepatic insulin resistance. These data may imply that nutrient excess coupled with OXPHOS defects determine the severity of NAFLD.

The mechanism by which enhanced OXPHOS increases the [NAD^+^]/[NADH] is yet unclear. It is plausible that enhanced cycling of NADH through complex I of the electron transport chain yields an increase in oxidized carrier intermediates. Loss of complex I in some studies [43], as well as our data from rotenone inhibition, substantiate that complex I function is critical for maintaining [NAD^+^]/[NADH]. Interestingly, mice with enhanced hepatic OXPHOS also have increased total NAD^+^ pools, possibly suggesting an alteration in NAD+ metabolism. Illustrating the complexity of this regulation, Karamanlidis et al., 2013 recently reported that complex I deficiency reduced the [NAD^+^]/[NADH] ratio but also perturbed the pool size. These observations suggest that the regulation of NAD^+^ and NADH is complex, presumably because it is governed by many variables. In our study, we did not observe any alteration in the rate limiting NAD^+^ synthetic genes. Ectopic expression of LRPPRC increases intracellular ATP, a co-factor critical for NAD^+^ synthesis. It is, thus, conceivable that enhanced OXPHOS increases cellular ATP, which in may in turn promote NAD^+^ synthesis. Coupled with enhanced cycling at complex I, this could explain the increase in both total pool size and [NAD^+^]/[NADH] ratio.

In our model which is protected against NAFLD and insulin resistance, respiration is coupled, indicative *bona fide* OXPHOS. Shulman and colleagues, however, showed that uncoupling cellular respiration in mice was associated with improved insulin sensitivity [44,45]. Our work illustrates that augmenting cellular respiration, whether coupled (that is, *bona fide* OXPHOS) or uncoupled, increases the ratio of [NAD^+^]/[NADH], implying a common mechanism whereby respiration-mediated control of NAD^+^, which governs complete oxidation of fatty acids and interdicts hepatic steatosis. This is important, because prior studies indicate that treatment with NAD^+^ precursors niacin or nicotinamide mononucleotide may have beneficial effects on fatty liver [46,47]. These effects are thought to be, in part, dependent on activation of the sirtuin family of deacetylases. Here we have demonstrated that cellular NAD^+^ is capable of promoting fatty acid oxidation in the absence of the mitochondrial sirtuin SIRT3. Moreover, we did not observe any effects on mitochondrial biogenesis, a downstream effect of nuclear sirtuin SIRT1 activation. NAD^+^, is a potent activator of TCA cycle enzymes, whereas NADH is an inhibitor. It is conceivable that increasing the ratio of [NAD^+^]/[NADH] or possibly increasing the absolute concentration of NAD^+^, might stimulate TCA enzymes and promote complete oxidation of fatty acids, independent of the activation of sirtuin proteins. Supporting a central role for NAD^+^ levels, increasing NAD^+^ in OXPHOS deficient hepatocytes rescued complete fatty acid oxidation in *Sirt3* knockout primary hepatocytes. Despite these observations, we cannot exclude other potentially salutary effects of increasing NAD^+^ that could enhance fatty acid oxidation in hepatocytes.

Other studies have manipulated mitochondrial content in the context of NAFLD, however, these manipulations have pleiotropic effects that confound interpretation. For example, ectopic expression of the transcriptional co-activator PGC-1β in mouse liver improved hepatic steatosis [34,35], but also induced enzymes involved in lipid export. Hence, it is unclear how significant OXPHOS *per se* was in this experimental model. More importantly, increased hepatic lipid secretion in this model might adversely affect cardiovascular health, an adverse change in serum lipids we did not observe in our mouse model which specifically augments OXPHOS. Other studies have evaluated the role of OXPHOS in fatty liver by ablating OXPHOS in mouse liver [48]. Ablation of OXPHOS in liver, however, is almost universally associated with reduced fat mass. Adiposity is a potent regulator of whole-body insulin sensitivity. It is, thus, possible that marked reductions in fat mass in models of impaired hepatic OXPHOS may offset adverse changes in liver. In our model of augmented OXPHOS, we saw a modest decline in weight. Even so, we suspect weight changes were not a major factor in our findings, as single and double transgenic mice had nearly identical weights, however, mitigation of NAFLD and insulin resistance was greatest only in double transgenic mice. Moreover, changes trending toward insulin sensitivity preceded changes in weight.

In summary, activation of hepatic OXPHOS increases NAD^+^ and interdicts NAFLD. The ratio of [NAD^+^]/[NADH] is dictated by cellular respiration and increases in NAD^+^ promote complete oxidation of fatty acids. These changes are ultimately associated with reduced cellular ROS, less activation of PKCε, and reduced inflammation, changes we believe promote whole-body and hepatic insulin sensitivity. OXPHOS-mediated control of NAD^+^ may prove a safe and effective means to interdict NAFLD.

## Supporting Information

S1 Fig Hemizygous expression of LRPPRC promotes mitochondrial transcription. (A) *Lrpprc*, mitochondrial polymerase (Polrmt), mitochondrial transcription factor A (Tfam), and mitochondrial transcription factor B2 (Tfb2m) and (B) mitochondrially encoded respiratory complex subunit gene expression in hemizygous liver specific LRPPRC transgenic (Tg/0) and control livers. Data are mean ± SEM. *p<0.05 by Student’s unpaired two-tailed t-test (A) or 2-way ANOVA (B).
(PDF)

S2 Fig LRPPRC increases respiratory complex activity. Individual electron transport chain complex activity of NADH-ubiquinone oxidoreductase (CI), ubiquinol-cytochrome c reductase (CIII), cytochrome c oxidase (CIV), and ATPase (CV) in control (LacZ) vs. LRPPRC replete samples, normalized to citrate synthase activity (CS). Citrate synthase activity was unchanged between groups. Data are mean ± SEM. p<0.05 by two-way ANOVA.
(PDF)

S3 Fig Effects of LRPPRC on OXPHOS are maintained after high-fat feeding. (A) Relative *Lrpprc* and (B) mitochondrially encoded respiratory subunit gene expression in livers of wild-type (WT) and double-hemizygous liver-specific *Lrpprc* transgenic mice fed a high-fat diet. (C) Protein expression in the same samples. Data are mean ± SEM. *p<0.05 by Student’s unpaired two-tailed t-test (A) or 2-way ANOVA (B).
(PDF)

S4 Fig Hemizygous expression of LRPPRC demonstrates moderate protection from NAFLD. (A) Representative H+E and (B) pathologic grading of wild-type (WT) and hemizygous liver-specific *LRPPRC* transgenic mice (Tg/0) fed a high-fat diet for 12 weeks, n = 8–10 for WT and n = 6 for Tg/0. Data are mean ± SEM. *p<0.05 by chi-square analysis.
(PDF)
