# Supplementary figures and images for: OXPHOS-Mediated Induction of NAD+ Promotes Complete Oxidation of Fatty Acids and Interdicts Non-Alcoholic Fatty Liver Disease
Source: PLoS One. 2015 May 1;10(5):e0125617. doi: 10.1371/journal.pone.0125617 (PMC4416931; doi:10.1371/journal.pone.0125617)

Figure S1

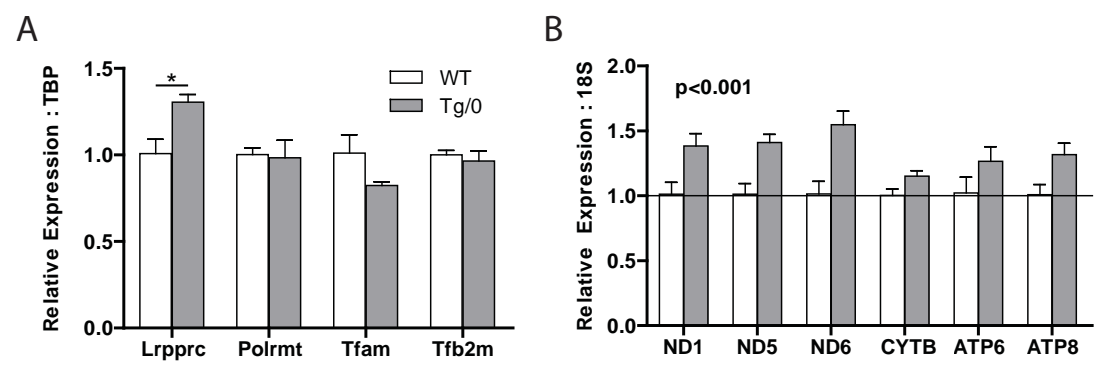

Supplement: S1 Fig — (A) Lrpprc, mitochondrial polymerase (Polrmt), mitochondrial transcription factor A (Tfam), and mitochondrial transcription factor B2 (Tfb2m) and (B) mitochondrially encoded respiratory complex subunit gene expression in hemizygous liver specific LRPPRC transgenic (Tg/0) and control livers. Data are mean ± SEM. *p<0.05 by Student’s unpaired two-tailed t-test (A) or 2-way ANOVA (B). (PDF) [file pone.0125617.s001.pdf]

Figure S2

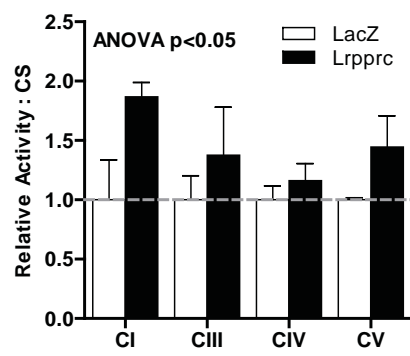

Supplement: S2 Fig — Individual electron transport chain complex activity of NADH-ubiquinone oxidoreductase (CI), ubiquinol-cytochrome c reductase (CIII), cytochrome c oxidase (CIV), and ATPase (CV) in control (LacZ) vs. LRPPRC replete samples, normalized to citrate synthase activity (CS). Citrate synthase activity was unchanged between groups. Data are mean ± SEM. p<0.05 by two-way ANOVA. (PDF) [file pone.0125617.s002.pdf]

Figure S3

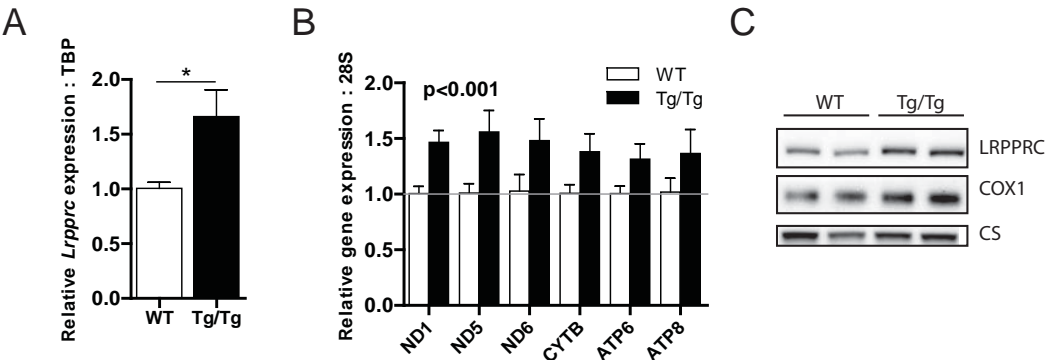

Supplement: S3 Fig — (A) Relative Lrpprc and (B) mitochondrially encoded respiratory subunit gene expression in livers of wild-type (WT) and double-hemizygous liver-specific Lrpprc transgenic mice fed a high-fat diet. (C) Protein expression in the same samples. Data are mean ± SEM. *p<0.05 by Student’s unpaired two-tailed t-test (A) or 2-way ANOVA (B). (PDF) [file pone.0125617.s003.pdf]

Figure S4

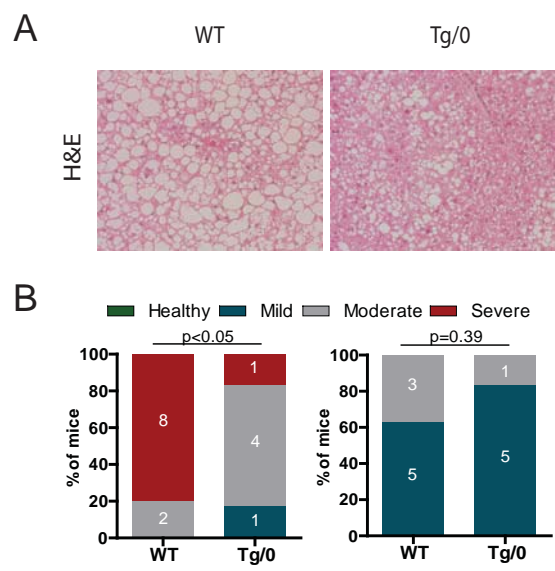

Supplement: S4 Fig — (A) Representative H+E and (B) pathologic grading of wild-type (WT) and hemizygous liver-specific LRPPRC transgenic mice (Tg/0) fed a high-fat diet for 12 weeks, n = 8–10 for WT and n = 6 for Tg/0. Data are mean ± SEM. *p<0.05 by chi-square analysis. (PDF) [file pone.0125617.s004.pdf]
